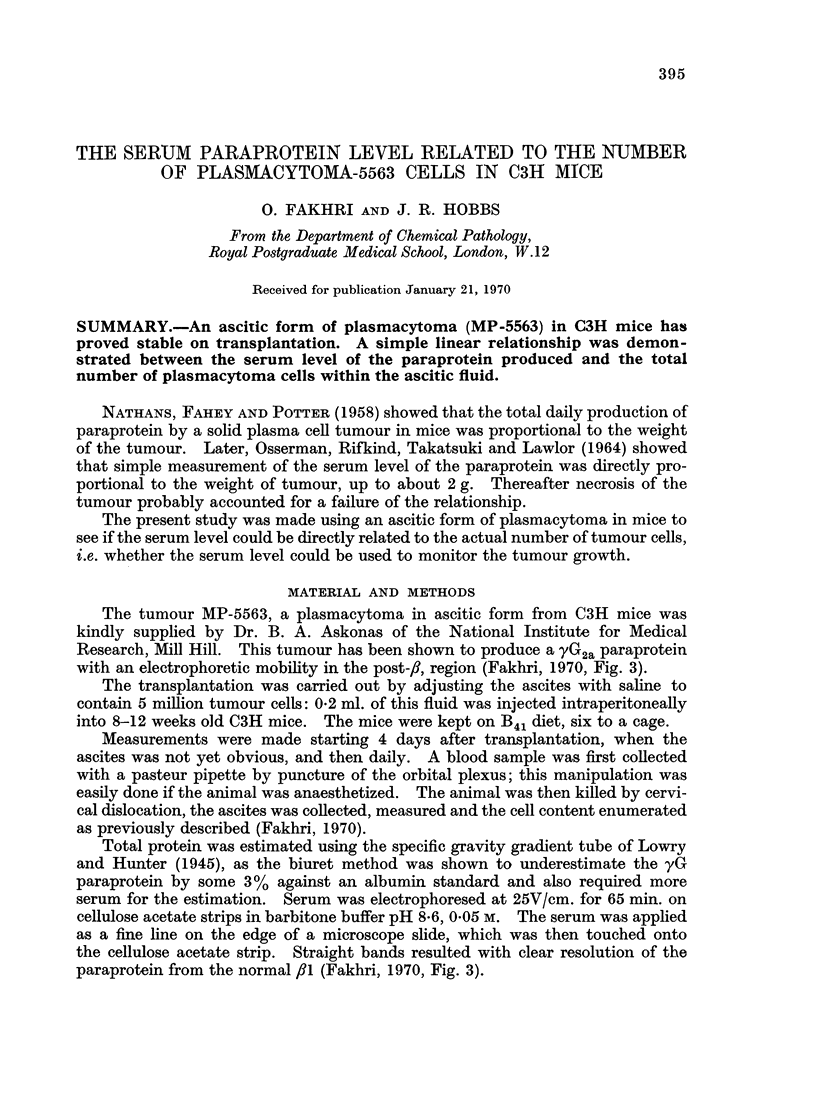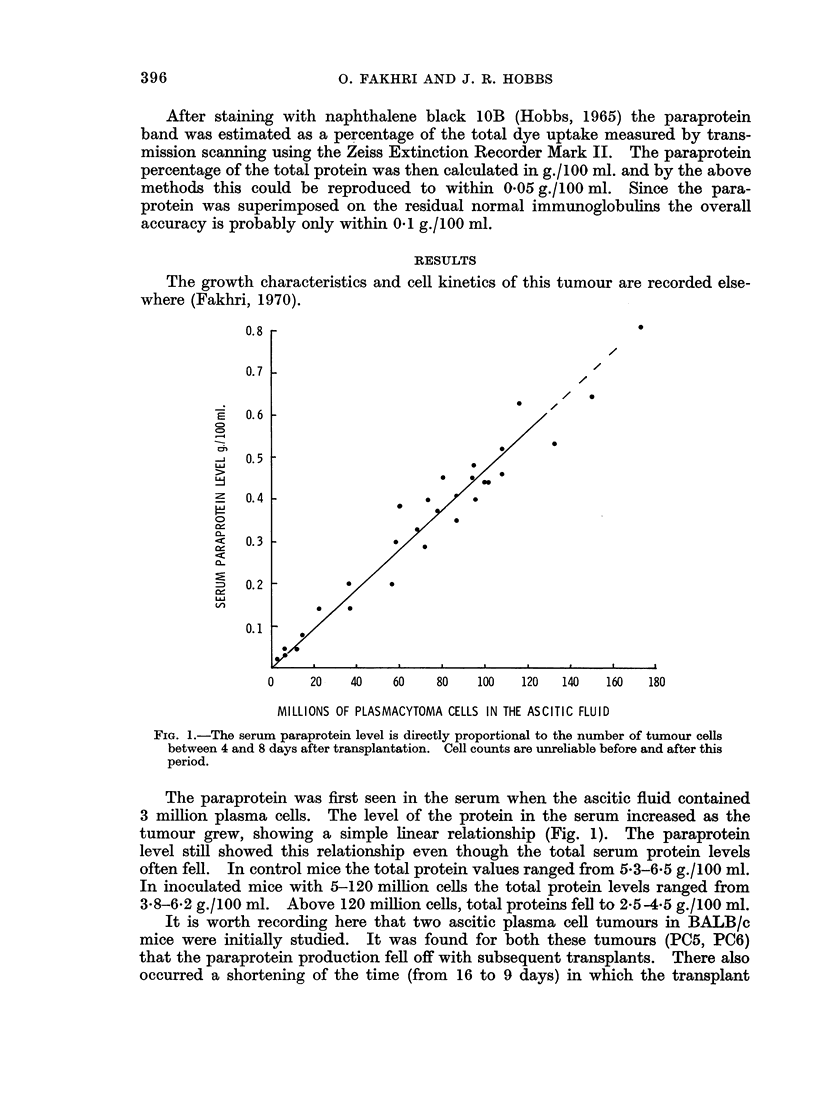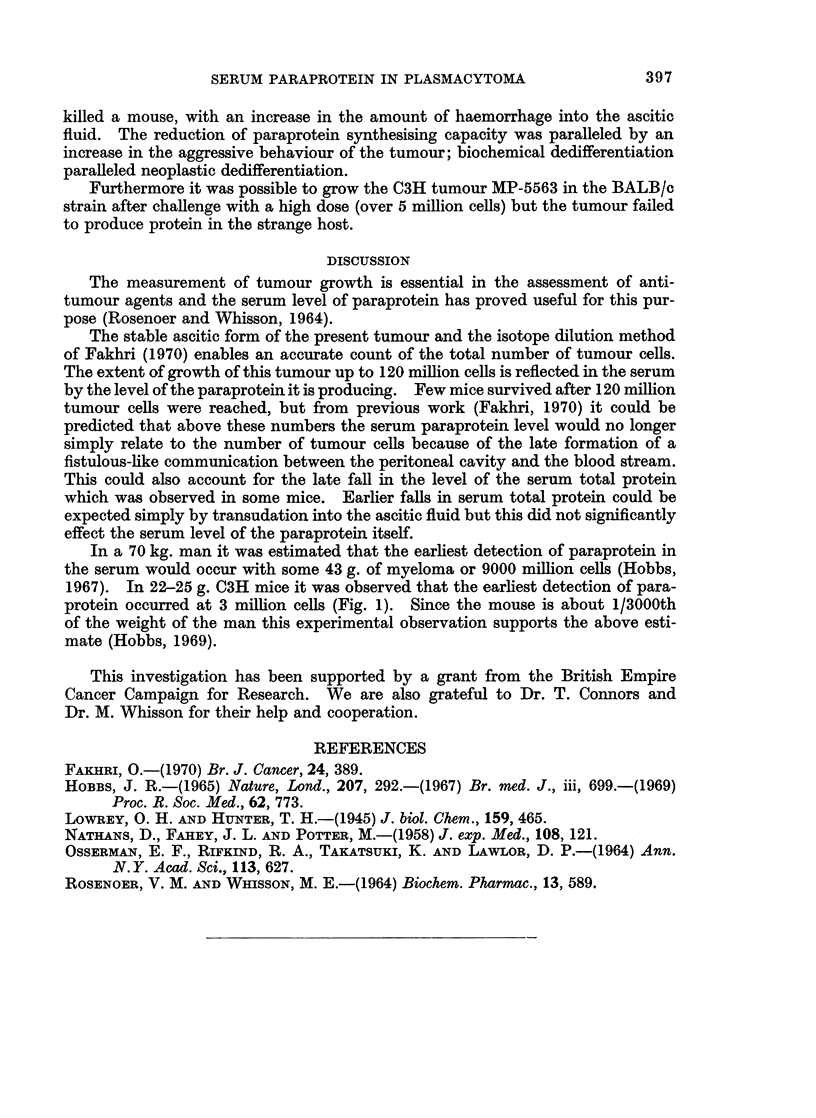# The Serum Paraprotein Level Related to the Number of Plasmacytoma-5563 Cells in C3H Mice

**DOI:** 10.1038/bjc.1970.46

**Published:** 1970-06

**Authors:** O. Fakhri, J. R. Hobbs

## Abstract

An ascitic form of plasmacytoma (MP-5563) in C3H mice has proved stable on transplantation. A simple linear relationship was demonstrated between the serum level of the paraprotein produced and the total number of plasmacytoma cells within the ascitic fluid.


### THE SERUM PARAPROTEIN LEVEL RELATED TO THE NUMBER OF PLASMACYTOMA-5563 CELLS IN C3H MICE

O. FAKHRI AND J. R. HOBBS

*From the Department of Chemical Pathology, Royal Postgraduate Medical School, London, W.12*

Received for publication January 21, 1970

**SUMMARY.—An ascitic form of plasmacytoma (MP-5563) in C3H mice has proved stable on transplantation. A simple linear relationship was demonstrated between the serum level of the paraprotein produced and the total number of plasmacytoma cells within the ascitic fluid.**

NATHANS, FAHEY AND POTTER (1958) showed that the total daily production of paraprotein by a solid plasma cell tumour in mice was proportional to the weight of the tumour. Later, Osserman, Rifkind, Takatsuki and Lawlor (1964) showed that simple measurement of the serum level of the paraprotein was directly proportional to the weight of tumour, up to about 2 g. Thereafter necrosis of the tumour probably accounted for a failure of the relationship.

The present study was made using an ascitic form of plasmacytoma in mice to see if the serum level could be directly related to the actual number of tumour cells, *i.e.* whether the serum level could be used to monitor the tumour growth.

### MATERIAL AND METHODS

The tumour MP-5563, a plasmacytoma in ascitic form from C3H mice was kindly supplied by Dr. B. A. Askonas of the National Institute for Medical Research, Mill Hill. This tumour has been shown to produce a $\gamma G_{2a}$ paraprotein with an electrophoretic mobility in the post-$\beta$, region (Fakhri, 1970, Fig. 3).

The transplantation was carried out by adjusting the ascites with saline to contain 5 million tumour cells: 0·2 ml. of this fluid was injected intraperitoneally into 8–12 weeks old C3H mice. The mice were kept on $B_{41}$ diet, six to a cage.

Measurements were made starting 4 days after transplantation, when the ascites was not yet obvious, and then daily. A blood sample was first collected with a pasteur pipette by puncture of the orbital plexus; this manipulation was easily done if the animal was anaesthetized. The animal was then killed by cervical dislocation, the ascites was collected, measured and the cell content enumerated as previously described (Fakhri, 1970).

Total protein was estimated using the specific gravity gradient tube of Lowry and Hunter (1945), as the biuret method was shown to underestimate the $\gamma G$ paraprotein by some 3% against an albumin standard and also required more serum for the estimation. Serum was electrophoresed at 25V/cm. for 65 min. on cellulose acetate strips in barbitone buffer pH 8·6, 0·05 M. The serum was applied as a fine line on the edge of a microscope slide, which was then touched onto the cellulose acetate strip. Straight bands resulted with clear resolution of the paraprotein from the normal $\beta 1$ (Fakhri, 1970, Fig. 3).

After staining with naphthalene black 10B (Hobbs, 1965) the paraprotein band was estimated as a percentage of the total dye uptake measured by transmission scanning using the Zeiss Extinction Recorder Mark II. The paraprotein percentage of the total protein was then calculated in g./100 ml. and by the above methods this could be reproduced to within 0·05 g./100 ml. Since the paraprotein was superimposed on the residual normal immunoglobulins the overall accuracy is probably only within 0·1 g./100 ml.

### RESULTS

The growth characteristics and cell kinetics of this tumour are recorded elsewhere (Fakhri, 1970).

Fig. 1.—The serum paraprotein level is directly proportional to the number of tumour cells between 4 and 8 days after transplantation. Cell counts are unreliable before and after this period.

The paraprotein was first seen in the serum when the ascitic fluid contained 3 million plasma cells. The level of the protein in the serum increased as the tumour grew, showing a simple linear relationship (Fig. 1). The paraprotein level still showed this relationship even though the total serum protein levels often fell. In control mice the total protein values ranged from 5·3–6·5 g./100 ml. In inoculated mice with 5–120 million cells the total protein levels ranged from 3·8–6·2 g./100 ml. Above 120 million cells, total proteins fell to 2·5-4·5 g./100 ml.

It is worth recording here that two ascitic plasma cell tumours in BALB/c mice were initially studied. It was found for both these tumours (PC5, PC6) that the paraprotein production fell off with subsequent transplants. There also occurred a shortening of the time (from 16 to 9 days) in which the transplant killed a mouse, with an increase in the amount of haemorrhage into the ascitic fluid. The reduction of paraprotein synthesising capacity was paralleled by an increase in the aggressive behaviour of the tumour; biochemical dedifferentiation paralleled neoplastic dedifferentiation.

Furthermore it was possible to grow the C3H tumour MP-5563 in the BALB/c strain after challenge with a high dose (over 5 million cells) but the tumour failed to produce protein in the strange host.

### DISCUSSION

The measurement of tumour growth is essential in the assessment of anti-tumour agents and the serum level of paraprotein has proved useful for this purpose (Rosenoer and Whisson, 1964).

The stable ascitic form of the present tumour and the isotope dilution method of Fakhri (1970) enables an accurate count of the total number of tumour cells. The extent of growth of this tumour up to 120 million cells is reflected in the serum by the level of the paraprotein it is producing. Few mice survived after 120 million tumour cells were reached, but from previous work (Fakhri, 1970) it could be predicted that above these numbers the serum paraprotein level would no longer simply relate to the number of tumour cells because of the late formation of a fistulous-like communication between the peritoneal cavity and the blood stream. This could also account for the late fall in the level of the serum total protein which was observed in some mice. Earlier falls in serum total protein could be expected simply by transudation into the ascitic fluid but this did not significantly effect the serum level of the paraprotein itself.

In a 70 kg. man it was estimated that the earliest detection of paraprotein in the serum would occur with some 43 g. of myeloma or 9000 million cells (Hobbs, 1967). In 22–25 g. C3H mice it was observed that the earliest detection of paraprotein occurred at 3 million cells (Fig. 1). Since the mouse is about 1/3000th of the weight of the man this experimental observation supports the above estimate (Hobbs, 1969).

This investigation has been supported by a grant from the British Empire Cancer Campaign for Research. We are also grateful to Dr. T. Connors and Dr. M. Whisson for their help and cooperation.

### REFERENCES

FAKHRI, O.—(1970) *Br. J. Cancer*, **24**, 389.

HOBBS, J. R.—(1965) *Nature, Lond.*, **207**, 292.—(1967) *Br. med. J.*, iii, 699.—(1969) *Proc. R. Soc. Med.*, **62**, 773.

LOWREY, O. H. AND HUNTER, T. H.—(1945) *J. biol. Chem.*, **159**, 465.

NATHANS, D., FAHEY, J. L. AND POTTER, M.—(1958) *J. exp. Med.*, **108**, 121.

OSSERMAN, E. F., RIFKIND, R. A., TAKATSUKI, K. AND LAWLOR, D. P.—(1964) *Ann. N.Y. Acad. Sci.*, **113**, 627.

ROSENOER, V. M. AND WHISSON, M. E.—(1964) *Biochem. Pharmac.*, **13**, 589.